# Differential paired stage-specific expression of *Babesia bovis* cysteine-rich GCC2/GCC3 domain family proteins (BboGDP) during development within *Rhipicephalus microplus*

**DOI:** 10.1186/s13071-022-05628-6

**Published:** 2023-01-17

**Authors:** Hala E. Hussein, Wendell C. Johnson, Massaro W. Ueti

**Affiliations:** 1 Department of Veterinary Microbiology and Pathology, Washington State University, Pullman, WA USA; 2 Animal Disease Research Unit, Agricultural Research Service (ARS), U.S. Department of Agriculture, Pullman, WA USA

**Keywords:** *Babesia bovis*, *Rhipicephalus microplus*, Cysteine-rich GCC2/GCC3 domains, qPCR

## Abstract

**Background:**

*Babesia bovis*, an intra-erythrocytic apicomplexan parasite, is one of the causative agents of bovine babesiosis, the most important tick-borne disease of cattle in tropical and subtropical regions. *Babesia bovis* has a complex life-cycle that includes sexual development within the tick vector. The development of a transmission blocking vaccine to control bovine babesiosis requires the identification of antigens displayed on the surface of the parasite during its development within tick vectors. Four *B. bovis* cysteine-rich GCC2/GCC3 domain protein (BboGDP) family members were previously identified and are differentially expressed as discrete pairs by either blood stages or kinetes. In this study we focused on two family members, BboGDP1 and -3, that are expressed by *Babesia* parasites during tick infection.

**Methods and results:**

Transcription analysis using quantitative PCR demonstrated that BboGDP1 and -3 were upregulated in in vitro-induced sexual stage parasites and during parasite development in the tick midgut. Moreover, protein expression analysis of BboGDP1 and -3 during the development of sexual stages in in vitro culture was consistent with their transcription profile. Live immunofluorescence analysis using polyclonal antibodies confirmed surface expression of BboGDP1 and -3 on in vitro-induced sexual stage parasites. In addition, fixed immunofluorescence analysis showed reactivity of anti-BboGDP1 and -3 polyclonal antibodies to kinetes.

**Conclusions:**

The collective data indicate that BboGDP1 and -3 are expressed by kinetes and on the surface of sexual stages of the parasites. The identified parasite surface membrane proteins BboGDP1 and -3 are potential candidates for the development of a *B. bovis* transmission blocking vaccine.

**Graphical Abstract:**

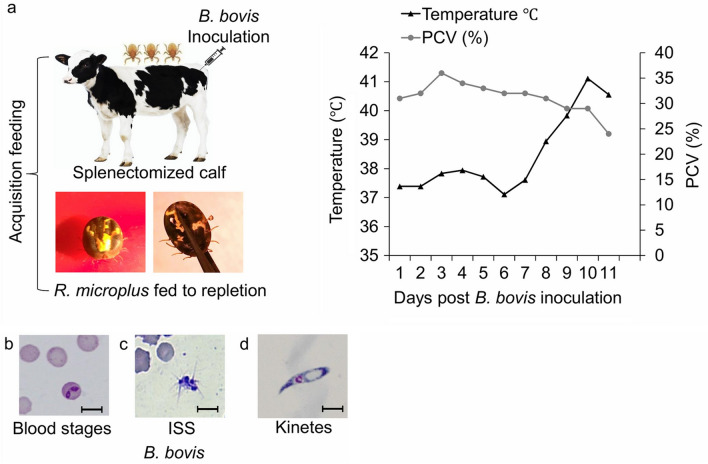

## Background

Bovine babesiosis is a tick-borne disease endemic in large parts of Australia, Africa, Asia, Europe and Latin America [1]. The disease is caused by the intra-erythrocytic parasites *Babesia bovis, Babesia bigemina* and *Babesia divergens*. *Babesia bovis* is transmitted primarily by the cattle fever tick, *Rhipicephalus microplus* [2, 3]*.* Bovine babesiosis is a significant health and economic issue for the cattle industry because of the high mortality and morbidity rates of infected animals. Bovine babesiosis control strategies, including acaricide treatment and live attenuated vaccines, are restricted due to increasing acaricide-resistant tick populations and by practical constraints of the live *Babesia* vaccines, such as possible reversion to virulence and the risk of tick transmission [4]. Despite safety concerns, several countries are still using live vaccines to mitigate acute infection and prevent mortality [4]. Therefore, the development of novel subunit vaccine approaches requires the identification of antigens critical for the completion of the parasite’s life-cycle [4].

*Babesia* parasites have a complex life-cycle that includes asexual replication in the mammalian host and sexual reproduction in the tick vector [2, 4]. Disruption of *B*. *bovis* development in the tick midgut (MG) would prevent transmission via tick vectors. The in vitro induction of *B*. *bovis* sexual stages using xanthurenic acid (XA) has enabled the identification of sexual stage-specific genes and gene families, such as the *hap2* gene [5], the cystine motif-rich six-cysteine (6-Cys) gene family [6], the *ccp* (cysteine-rich polycomb-like protein) gene family [7], calcium-dependent protein kinase 4, tubulin-tyrosine ligase and methyltransferase [8]. These genes encode proteins that may be important candidates for developing an effective drug or vaccine to control bovine babesiosis.

*Babesia bovis* cysteine-rich grip and coiled-coil domain containing 2 and 3 (GCC2/GCC3) proteins (BboGDP) are encoded by a small gene family that is conserved in malaria and other apicomplexan parasites [9]. In a closely related parasite, *Plasmodium*, Cysteine Repeat Modular Proteins (PCRMP) are four conserved proteins containing a number of motifs implicated in host-parasite interactions [9]. The PCRMP family is expressed as pairs that function during different stages of the parasite’s life-cycle [10, 11]. BboGDP1 (BBOV_III011730), BboGDP2 (BBOV_III011740) BboGDP3 (BBOV_IV006250) and BboGDP4 (BBOV_IV006260) were previously identified as large, predicted surface proteins with multiple transmembrane domains containing motifs with a unique combination of protein-binding motifs, including cysteine-rich regions and epidermal growth factor-like domains [12]. BboGDP were shown to be upregulated as discrete pairs by both *B. bovis* blood stages and kinetes [12]. Studies on BboGDP revealed that BboGDP1 and -3 were upregulated in kinetes, whereas BboGDP2 and -4 were upregulated in blood stages [12, 13], supporting the concept that BboGDP genes may be important for infection of the mammalian host and essential for parasite transmission through the invertebrate host [12]. The goal of this study was to understand the expression profile of BboGDP1 and -3 during parasite development of sexual stages and to identify promising candidates implicated in *Babesia* parasite-tick interactions that may facilitate parasite transmission.

## Methods

### Cattle, ticks and parasite cultures

**Fig. 1:**
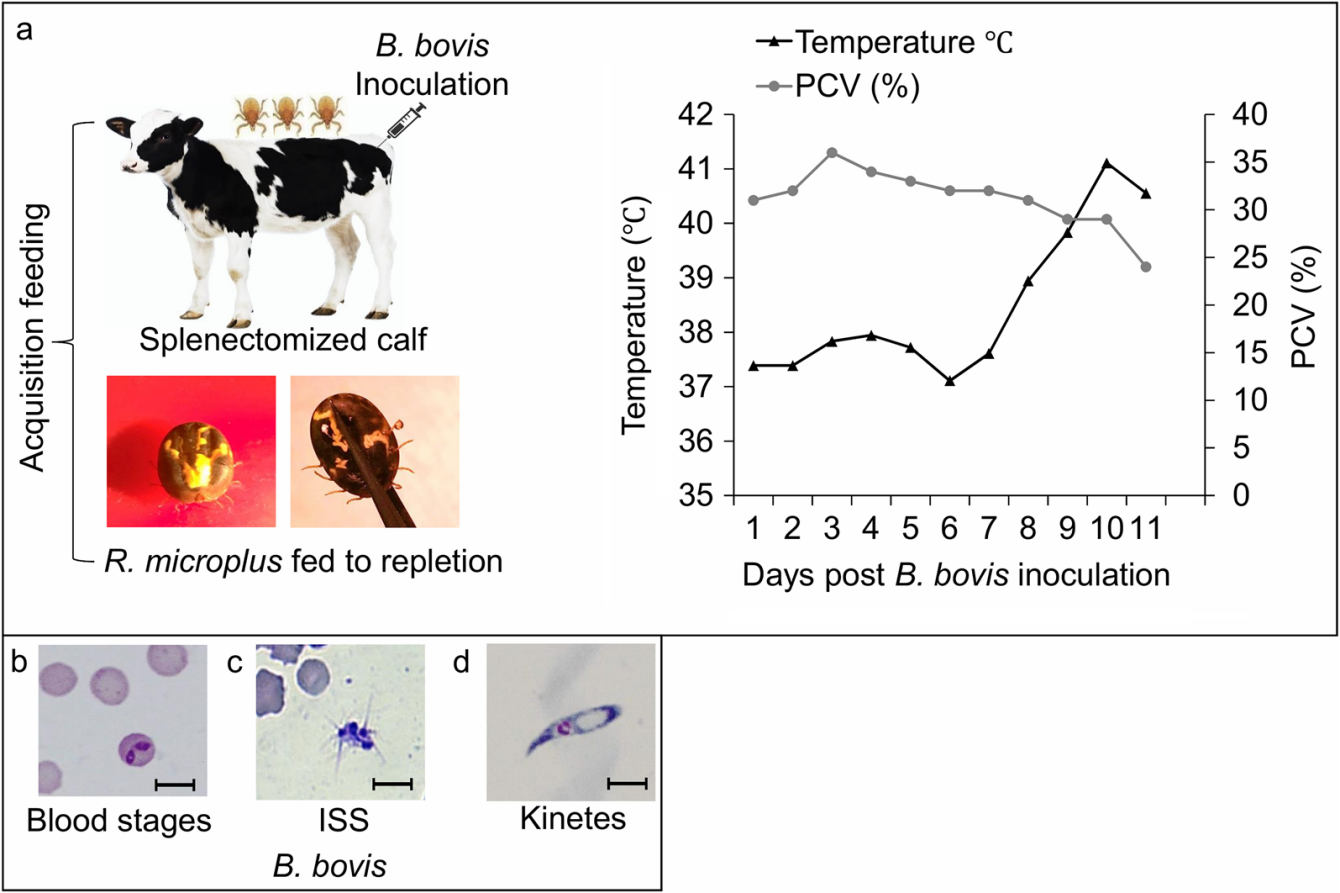
Acute infection of *Babesia bovis*-infected calf*.* **a** Inoculation of *B. bovis* and acquisition feeding; calf clinical signs (PCV reduction and temperature rise), **b** *B. bovis* blood stages, **c** ISS, **d** kinetes. ISS, Induced sexual stages; PCV, packed cell volume

A splenectomized 4-month-old male Holstein calf that tested negative for *B. bovis* by PCR [2] and complement-enzyme linked immuno sorbent assay (cELISA) [14] was used in this study. The *Rhipicephalus microplus* La Minita tick strain was used as previously described [2, 15]. The calf was inoculated intravenously with *B. bovis* S_74_T_3_Bo strain stabilate containing approximately 1 × 10^7^ *B. bovis*-infected erythrocytes [2, 12] to synchronize the peak of parasitemia with female tick repletion. The infected calf was monitored daily for the presence of *B. bovis* in the peripheral blood and clinical signs of babesiosis (Fig. 1a). The animal was maintained according to protocols approved by the University of Idaho Institutional Animal Care and Use Committee (IACUC #2018–16).

### *Babesia bovis* blood stages

Defibrinated blood was collected from the calf 11 days post-*B*. *bovis* inoculation. Blood was collected into flasks containing glass beads and shaken to prevent blood coagulation. Red blood cells (RBCs) from defibrinated blood were washed with Puck’s Saline G to remove white blood cells. Some of the washed infected RBCs were pelleted by centrifugation (3000 rpm, 10 min, 4 °C) and suspended in TRIzol (Thermo Fisher Scientific, Waltham, MA, USA). For the establishment of *B*. *bovis *in vitro culture, infected RBCs were placed into flasks with culture medium as previously described [16] and incubated at 3% O_2_ and 5% CO_2_. After the in vitro incubation of *B. bovis* blood stages, a portion of the *B. bovis* cultures were used to induce sexual stages; the other portion was suspended in TRIzol and stored at − 20 °C.

### In vitro induction of *B. bovis* sexual stages

To induce the sexual stages of this parasite, in vitro-cultured *B*. *bovis*-infected erythrocytes (Fig. 1b) were suspended in a medium containing 100 μM XA (Sigma-Aldrich, St. Louis, MO, USA) at 26 °C with 5% CO_2_ as previously described [5]. Induced in vitro sexual stage parasites (Fig. 1c) were isolated at 24 h post-induction by differential centrifugation at 400 *g* for 1 min. The supernatant was recovered, and the sexual stages pelleted by centrifugation at 2000 *g* for 5 min. A portion of the induced sexual parasites was suspended in TRIzol and stored at -20 °C; another portion was used for immunofluorescence assays.

### *Babesia bovis*-infected engorged tick MG

Replete female ticks were collected, washed in tap water, dried and incubated at 26 °C and 92% relative humidity. During the development of *B*. *bovis* within the tick MG, six engorged ticks were removed daily from the incubator and dissected, for 6 consecutive days. Each individual MG was placed into 1 ml of TRIzol® reagent (Thermo Fisher Scientific) and stored at − 20 °C.

### *Babesia bovis* kinetes

Hemolymph was sampled from incubated females individually on day 8 post-tick incubation to select ticks with a high number of kinetes, as previously described [2, 12]. A distal leg segment was removed (Fig. 1a), and a drop of exuded hemolymph was placed onto a glass slide and stained with Giemsa stain (Fig. 1d), as previously described [12]. Kinetes were collected by extraction of hemolymph-containing fluid with negative pressurized capillary tubing, pooled and concentrated by centrifugation (4000 *g*, 2 min) [17] to be processed for immunofluorescence assays.

### RNA extraction and complementary DNA synthesis

Total RNA was extracted from *B*. *bovis*-infected blood, sexual stages and tick gut samples in TRIzol reagent (Invitrogen, Thermo Fisher Scientific) according to manufacturer’s protocol and the RNA pellets subsequently suspended in 20 µl DEPC-treated water (Invitrogen, Thermo Fisher Scientific). RNA samples were treated with DNase I (Invitrogen, Thermo Fisher Scientific) following the manufacturer’s protocol to remove contaminating genomic DNA and quantified by spectrophotometry on a NanoDrop spectrophotometer (Thermo Fisher Scientific). The removal of genomic DNA was confirmed by PCR assays that targeted Rap1 as previously described [18] using non-reverse transcribed samples. Complementary DNA (cDNA) was synthesized from 150 ng of total RNA of each sample with a Superscript® First-strand cDNA synthesis kit (Invitrogen, Thermo Fisher Scientific) following the manufacturer’s protocol.

### Quantitative PCR assay

The expression pattern of BboGDP1-4 was examined by quantitative PCR (qPCR). Specific primers for each gene were designed using the PrimerQuest® Tool (Integrated DNA Technologies, Coralville, IA, USA) (Table 1) following recommended guidelines for qPCR primer design. Standard PCR was performed to amplify all target genes from cDNA samples using the primers listed in Table 1. PCR cycling conditions consisted of 95 °C for 3 min, followed by 35 cycles of 95 °C for 30 s, 55 °C for 30 s, and 72 °C for 30 s, with a final extension at 72 °C for 5 min. The PCR products were separated and visualized by 1% agarose gel electrophoresis. PCR amplicons were cloned into PCR 2.1-TOPO® (Thermo Fisher Scientific) and submitted for sequencing (Eurofins MWG Operon, Louisville, KY, USA). Standard curves were generated for each gene using specific quantities of each plasmid. For the normalization of qPCR data, *B*. *bovis actin* (BBOV_IV009790) was evaluated and used as a parasite reference gene candidate. CFX Manager™ software (Bio-Rad Laboratories, Hercules, CA, USA) [19] was used to examine the stability of expression of the reference gene. The qPCR assays for the genes of interest and reference gene were performed in a CFX96™ Real-Time PCR Detection System (C1000 Touch™ Thermal Cycler; Bio-Rad Laboratories) using the SsoFast™ EvaGreen® Supermix Kit (Bio-Rad Laboratories). The cycling conditions consisted of an initial denaturation at 95 °C for 2 min, followed by 40 cycles of 95 °C (denaturation) for 15 s and 55 °C for 30 s (annealing). The reactions were performed in triplicate in a 20-μl reaction volume containing 300 nM of each primer and 2 μl of ½0 dilution of cDNA as template. CFX Manager™ software (Bio-Rad Laboratories) was used to analyze the qPCR data. Amplification efficiency was evaluated to determine the sensitivity of the qPCR for each gene. Relative expression was calculated by dividing each gene’s detected expression by the detected *actin* expression within each time point. Pairwise differences of time were tested with Tukey or Tukey–Kramer adjustment [8].

**Table 1:** Gene identification and primer sets of *Babesia bovis* genes of interest used for quantitative reverse transcriptase-PCR

*B. bovis* gene	Locus tag	Forward primers (5′-3′)	Reverse primers (5′-3′)	Size^a^
BboGDP1	BBOV_III011730	TGTGGATCACGAGCTGAGTC	CGGTGTTTCCATGGATTAGG	113
BboGDP2	BBOV_III011740	TGGATGAAGGTGACAAGTGC	TGTGGTACGGCAAAGAGTGA	193
BboGDP3	BBOV_IV006250	AAACGCTGCGCAAAAATAGT	AAGTACCAGCTTCGCAAGGA	120
BboGDP4	BBOV_IV006260	ATTGGCAAAGCCACTAATCG	CGTGAAGAAGATGCAGACCA	102
Actin	BBOV_IV009790	GAACGCCTGTCATTCGAGTT	GAAGCAAGCACCTTTCCAAC	111

BboGDP1–4, *Babesia bovis* GCC2/GCC3 domain-containing proteins

^a^Amplicon size in base pairs

### Antibody production

Polyclonal antibodies against BboGDP1 and -3 were produced as previously described [12] by immunizing rabbits with synthetic peptides. For each protein, three synthetic peptides predicted to be surface-exposed B-cell epitopes using a proprietary algorithm were synthesized (BioSynthesis, Inc., Lewisville, TX, USA) and used to immunize rabbits (Table 2).

**Table 2:** Peptides used to generate rabbit-specific antibodies against *B. bovis* proteins BboGDP1 and BboGDP3

*B. bovis* protein	*B. bovis* protein ID	Accession number	Peptide 1	Peptide 2	Peptide 3
BboGDP1	BBOV_III011730	EDO08725	EKERRDVEELERKLEC	SDEDIEKERRDVEELC	RDERKQLVYYGNSKPC
BboGDP3	BBOV_IV006250	EDO06984	DQSQRTPSRRDKLPA	LHNEAKFANHRHQKT	NRPSKIESCETNTWR

### Detection of surface-exposed proteins on *B. bovis* induced sexual stages

Live *B. bovis* parasites from blood stages and induced sexual stage cultures were washed in 3% normal goat serum in phosphate-buffered saline (PBS). Cells were then incubated for 1 h with a 1:100 dilution of primary antibodies (anti-BboGDP1 or anti- BboGDP3) in blocking solution. The cells were then washed twice in PBS by centrifugation at 400* g *and incubated for 30 min with 1:1000 goat anti-rabbit immunoglobulin G (IgG) Alexa Fluor 647 secondary antibody (Thermo Fisher Scientific) diluted with 10% normal goat serum. The cells were again washed twice with PBS and incubated with the nucleic acid stain Hoechst 33342 (Thermo Fisher Scientific) for 30 min. Finally, the cells were washed twice with PBS, and air dried on slides. Identically produced negative controls were performed using pre-immune (PI) rabbit serum as the primary antibodies. All samples were independently visualized by fluorescent microscopy using a Leica microscope equipped with LAS-X software (Leica Microsystems GmbH, Wetzlar, Germany).

### Evaluation of BboGDP expression by kinete stages

Fixed immunofluorescence assays (IFA) were used to evaluate kinete expression of BboGDP1 and -3. *Babesia bovis* kinete slides were prepared from infected *B. bovis* hemolymph as described above and washed 3 times with 10% normal goat serum in PBS. A 5-μl drop of suspended kinetes was added to wells of Teflon-printed glass slides (Electron Microcopy Sciences, Hatfield, PA, USA), and the slides were then air-dried and stored at − 80 °C. To perform the IFA, slides for *B. bovis* kinetes were placed into a desiccator jar for 20 min, fixed in cold acetone for 1 min and air-dried. A blocking solution of 10% normal goat serum in PBS was added, and the slides were incubated at 37 °C for 15 min in a humidified chamber. The slides were probed with a 1:100 dilution of primary antibodies (anti-BboGDP or anti-BboGDP3), rabbit pre- and post-immune sera in blocking solution and incubated for 1 h as before. The slides were then washed 3 times in cold PBS for 10 min, following which a 1:1000 goat anti-rabbit IgG Alexa Fluor 647 secondary antibody (Thermo Fisher Scientific) in blocking buffer was added to the wells, and the slides were incubated for 30 min as before. The slides were washed twice with PBS for 10 min, once with distilled water for 5 min and air-dried, then the nuclei were stained with 4,6-diamidino-2-phenylindole dihydrochloride (DAPI) (Thermo Fisher Scientific). The Slides were examined and visualized independently by fluorescent microscopy using a Leica microscope equipped with LAS-X software (Microsystems GmbH).

## Results

### BboGDP gene expression

Quantitative PCR was used to analyze the transcription pattern of BboGDP genes in blood collected from an acutely infected animal, in non-induced culture 0 h, in culture induced with XA at 24 h and in tick-specific stages from individual engorged tick MG samples collected from *B*. *bovis-*infected females. *Babesia bovis actin* was used as a reference gene for data normalization. The transcription levels of all target genes were normalized to the *actin* expression level. The melt curve analyses showed the absence of primer-dimers and nonspecific amplification for all tested genes; the efficiency of amplification ranged between 92% and 102%.

**Fig. 2:**
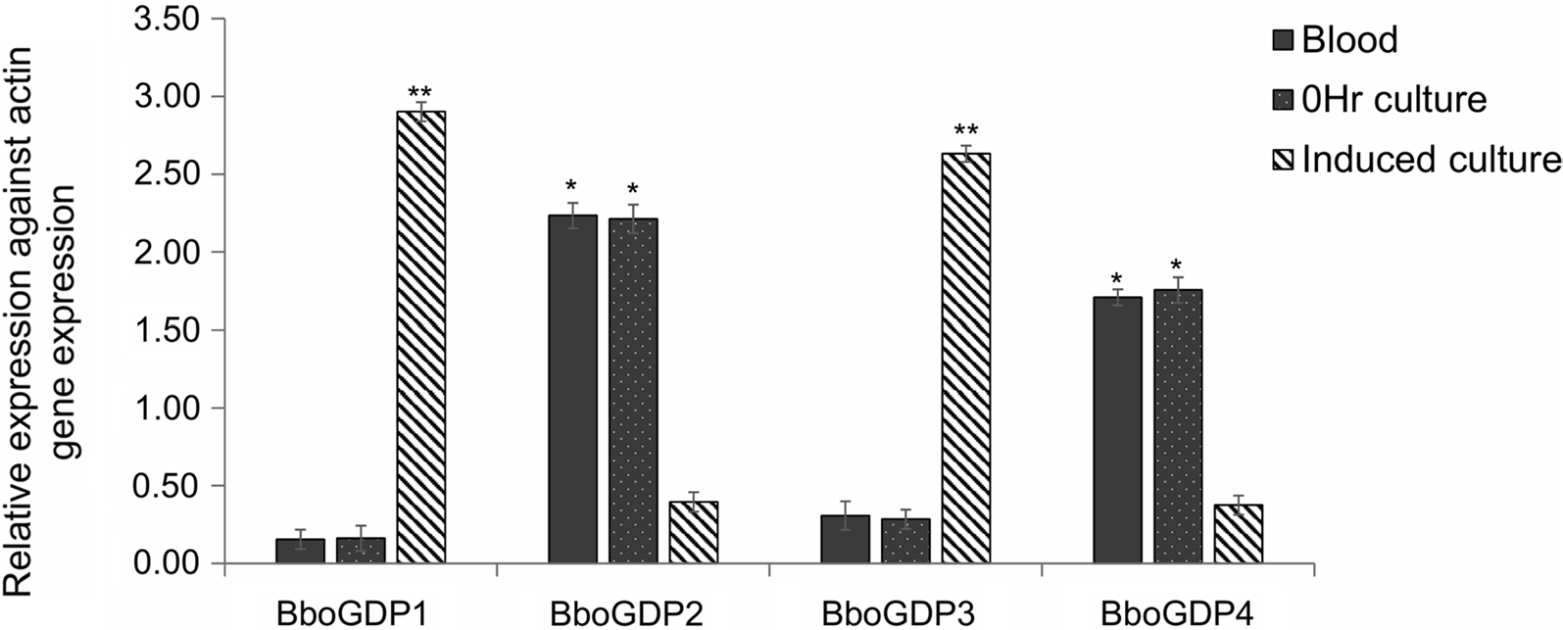
Transcriptional analysis of *B. bovis* BboGDP genes in blood from an acutely infected animal, cultured blood stages and induced sexual stages. The data represent the mean of three experiments, each containing three technical replicates. Asterisk indicates statistical pairwise differences between time points (*) (*P* < 0.05), (**) (*P* < 0.001). BboGDP1–4, *B. bovis* GCC2/GCC3 domain proteins 1–4

The data demonstrated that BboGDP1 and -3 were significantly upregulated in induced sexual stages when compared to blood stages (*P* < 0.001) (Fig. 2). In contrast, BboGDP2 and -4 were significantly downregulated in induced sexual stages when compared to blood stages (*P* < 0.05) (Fig. 2).

**Fig. 3:**
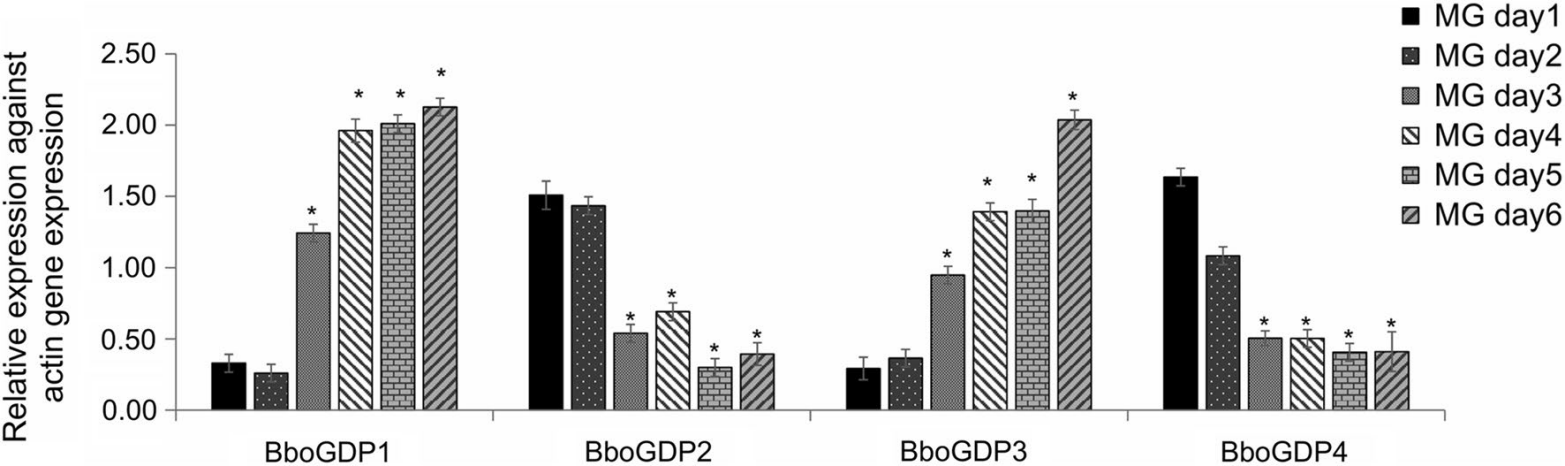
Transcriptional analysis of *B. bovis* BboGDP genes in specific tick stages from individual engorged female tick MG samples collected for 6 consecutive days after incubation (MG day1 to day6). The data represent the mean of three experiments, each containing three technical replicates. Asterisk indicates statistical pairwise differences between time points at *P < 0.05. MG, Midgut

Gene expression during MG infection demonstrated that BboGDP1 and -3 were upregulated in specific tick stages at days 3 to 6 when compared with days 1 and 2 (*P* < 0.05) (Fig. 3), while BboGDP2 and -4 expression were reduced at days 3 to 6 when compared with days 1 and 2 (*P* < 0.01) (Fig. 3). The results represent the mean of three experiments, each containing three technical replicates. Taken together these results demonstrated differential paired stage-specific regulation of BboGDP where BboGDP1 and -3 were significantly upregulated in induced sexual stages of the parasites when compared to blood stages as well as during parasite development within the tick MG.

### Protein expression by *B. bovis* blood stages, induced sexual stages and kinetes

**Fig. 4:**
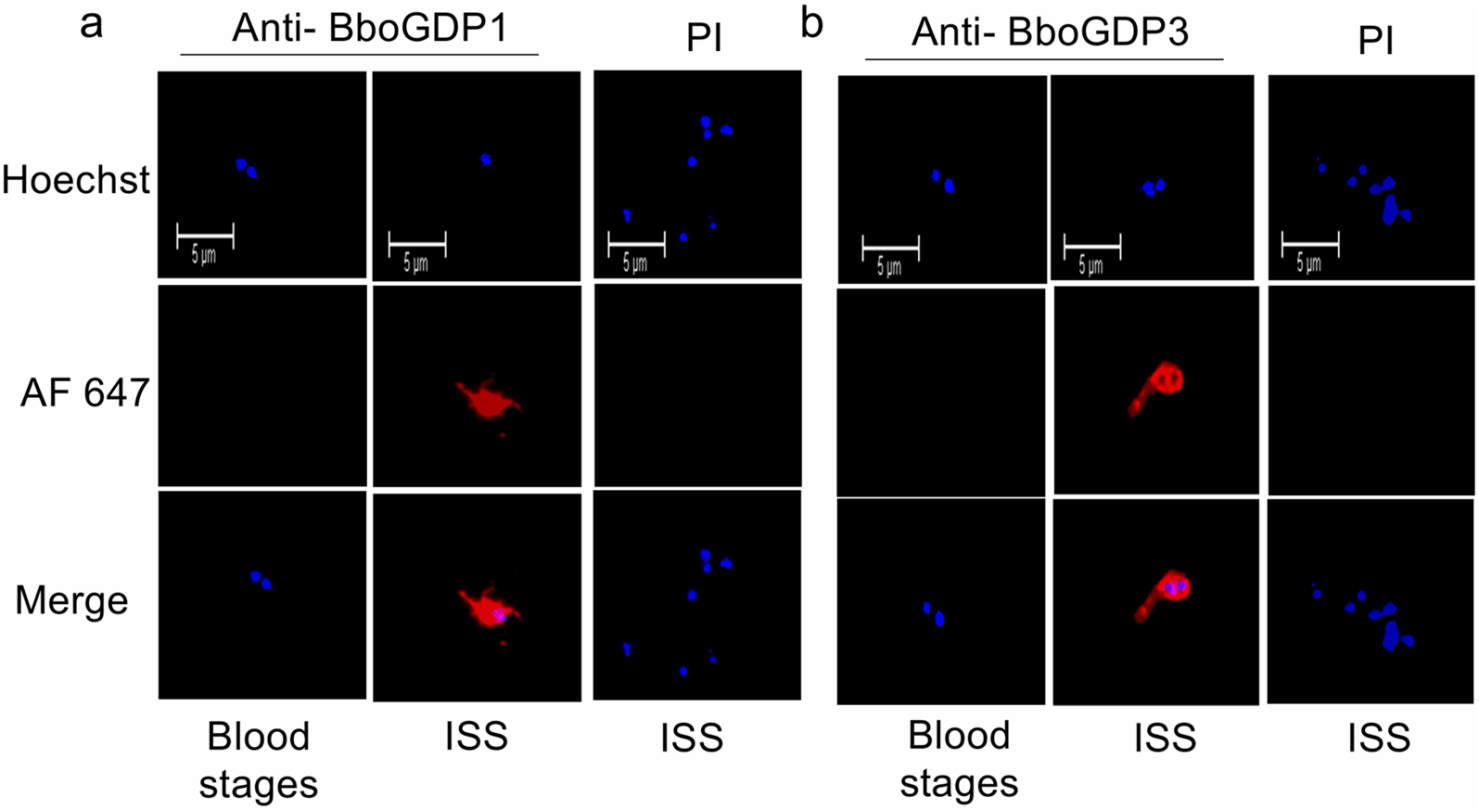
Live immunofluorescence detection of BboGDP1 and BboGDP3 expression on the surface of induced *B. bovis* extracellular parasites. **a** *B. bovis* blood stages and induced sexual stages incubated with anti-BboGDP1 and goat anti-rabbit tagged with AF 647 and stained with Hoechst; the negative control was PI rabbit serum as the primary antibody and stained with Hoechst. **b** *B. bovis* blood stages and induced sexual stages incubated with anti-BboGDP3 and goat anti-rabbit tagged with AF 647 and stained with Hoechst; the negative control was PI rabbit serum as the primary antibody and stained with Hoechst. Scale bars: 5 μm. AF 647, Alexa Fluor 647 stain; DAPI, 4,6-diamidino-2-phenylindole dihydrochloride; PI, pre-immune

Anti-BboGDP1 polyclonal antibody reacted to live parasite in vitro-induced sexual stages but was undetectable to *B. bovis* blood stages (Fig. 4a). Similarly, anti-BboGDP3 polyclonal antibody reacted to live parasite from in vitro-induced sexual stages and was undetectable to *B. bovis* blood stages (Fig. 4b). Live IFA indicated that BboGDP1 and -3 were proteins expressed on the surface of *B. bovis* sexual stages.

**Fig. 5:**
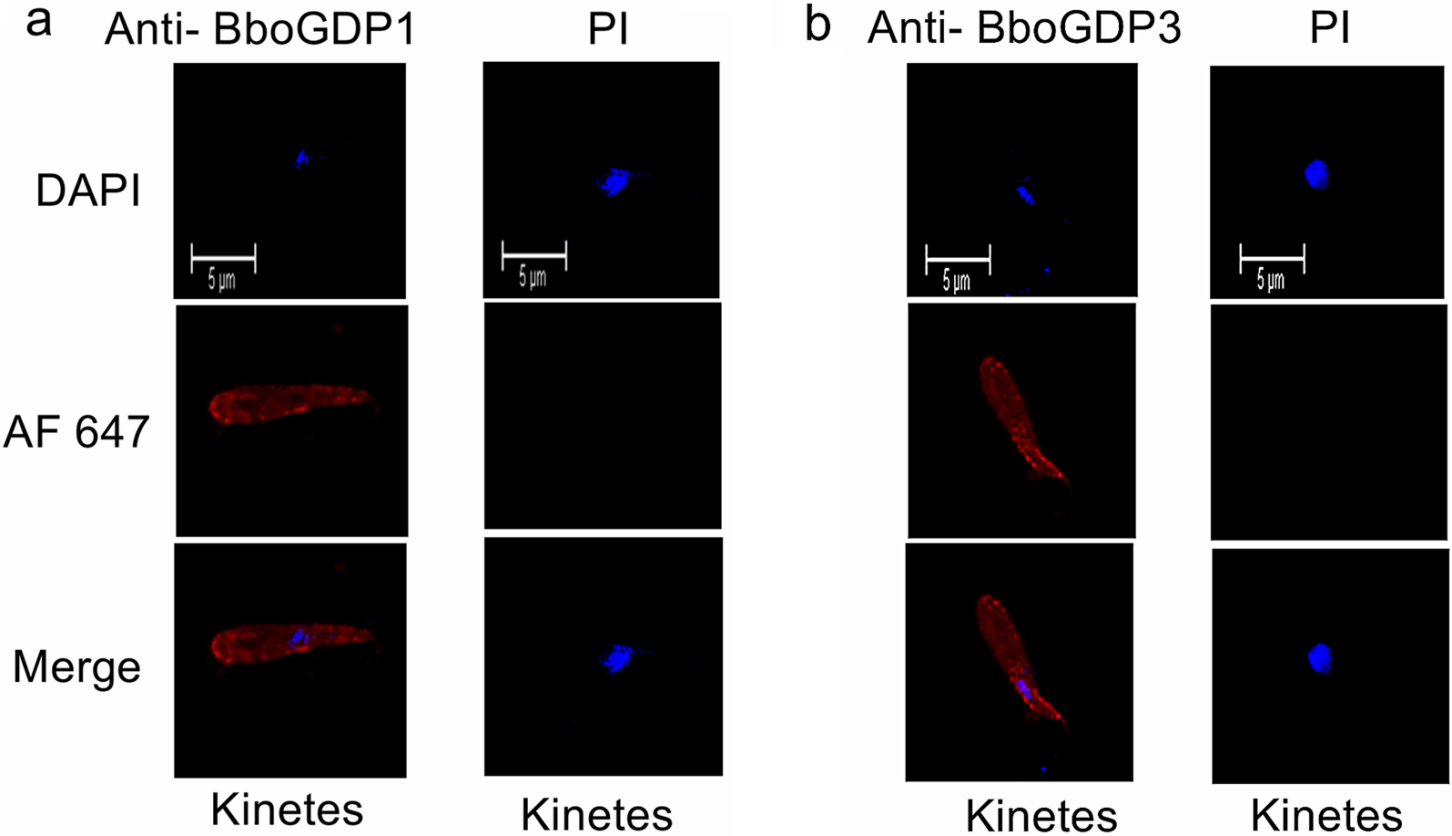
Immunofluorescence detection of BboGDP1 and BboGDP3 by *B. bovis* kinete stages.** a** *B. bovis* kinetes incubated with anti-BboGDP1 and goat anti-rabbit tagged with AF 647 and stained with DAPI; the negative control was PI rabbit serum as the primary antibody and stained with DAPI.** b** *B. bovis* kinetes incubated with anti-BboGDP3 and goat anti-rabbit tagged with AF 647 and stained with DAPI; the negative control PI rabbit serum was the primary antibody and stained with DAPI. Scale bars: 5 μm

Using fixed IFA, we demonstrated that anti-BboGDP1 (Fig. 5a) and anti-BboGDP3 (Fig. 5b) polyclonal antibodies reacted to kinetes, indicating that both proteins were expressed by *B. bovis* development with tick hemolymph. We were unable to determine if BboGDP1 and -3 were surface exposed on kinetes due to the lack of methods to isolate intact live parasites from the tick hemolymph.

**Table 3:** National Center for Biotechnology Information conserved domain search of *B. bovis* BboGDP

*B. bovis* protein ID	Accession number	Length	Signal peptide	Transmembrane domain	Glycosylphosphatidylinositol-anchored proteins	Domains (NCBI conserved domain search)
BBOV_III011730	EDO08725	2721 aa	No	Yes- 8	No	*cd00185* Location: 1054 to 1131—TNFRSF *pfam07699* Location: 1331 to 1377—GCC2/GCC3 *cl22855* Location: 1218 to 1287—TNFRSF
BBOV_III011740	EDO08726	2678 aa	Yes	Yes- 8	No	*pfam03302* Location: 1115 to 1383—VSP *cd00185* Location: 1541 to 1609—TNFRSF *pfam07699* Location: 1439 to 1482—GCC2/GCC3 *cl22855* Location: 1329 to 1452—TNFRSF
BBOV_IV006250	EDO06984	3719 aa	Yes	Yes-10	No	*cd00185* Location: 2205 to 2249—TNFRSF *pfam07699* Location: 2254 to 2302—GCC2/GCC3 *cl22855* Location: 2205 to 2319—TNFRSF
BBOV_IV006260	EDO06985	2948 aa	Yes	Yes- 8	No	*cd00185* Location: 1764 to 1798—TNFRSF *pfam07699* Location: 1759 to 1798—GCC2/GCC3 *cl22855* Location: 1884 to 1963—TNFRSF

*GCC2/GCC3* Gripped coiled-coil domain,* NCBI* National Center for Biotechnology Information,* TNFRSF* tumor necrosis factor super family,* VSP Giardia* variant-specific surface protein

## Discussion

The production of effective transmission blocking vaccines to control the spread of *Babesia* parasites depends on the exploration of sexual reproduction within the tick vector, although molecular mechanisms of this process remain largely unclear. In the present study, we examined the gene expression of BboGDP1 and -3 associated with developing sexual stages in induced in vitro cultures and MG of replete *R*. *microplus* females fed on *B*. *bovis*-infected calves.

BboGDP have been previously identified as large, predicted surface proteins with multiple transmembrane domains containing motifs that are conserved within families of cysteine-rich proteins, and which have a unique combination of protein-binding motifs, including cysteine-rich regions and epidermal growth factor-like domains (Table 3) [12]. BboGDP are well-conserved in *Plasmodium* Cysteine Repeat Modular Proteins (PRMP1-4) [9]. PCRMP1-4 are differentially expressed during different stages of the malaria parasite’s life-cycle. The expression pattern and structural features of the PCRMPs suggest a variety of roles mediating host–parasite interactions throughout the parasite life-cycle [9, 10, 20].

The data in the present study demonstrated gene expression of BboGDP1 and -3 in in vitro-induced sexual stage parasites and during parasite development within the tick MG. Previous studies have shown BboGDP1 and -3 to have higher levels of transcripts in kinetes, while BboGDP2 and -4 had higher levels of transcripts in the blood stages [12, 13]. A comparative bioinformatic analysis showed that *Babesia bigemina* GCC2/GCC3 domain proteins (BBBOND_0404220, BBBOND_0404210, BBBOND_0208320 and BBBOND_0208300) orthologous to BboGDP displayed similar domain structures with high amino acid identity of 57%, 54%, 42% and 45%, respectively (Table 4). A previous study showed that the transcription pattern of *B. bigemina* BBBOND_0208320, orthologous to *B. bovis* BboGDP3, had higher levels of transcripts in *B. bigemina* kinetes, supporting the notion that these proteins play distinct roles in the *Babesia* life-cycle within the tick vector [12].

**Table 4:** BboGDP sequence identities (%) between *Babesia bovis* and *Babesia bigemina*

*B. bovis* protein ID	*B. bovis* protein accession number	*B. bigemina* protein ID	*B. bigemina* protein accession number	Identity (%)	*B. bovis* function annotation
BBOV_III011730	EDO08725	BBBOND_0404220	XP_012770120	57	GCC2 and GCC3 domain-containing protein
BBOV_III011740	EDO08726	BBBOND_0404210	XP_012770119	54	GCC2 and GCC3 domain-containing protein
BBOV_IV006250	EDO06984	BBBOND_0208320	XP_012767864	42	GCC2 and GCC3 domain-containing protein
BBOV_IV006260	EDO06985	BBBOND_0208300	XP_012767862	45	GCC2 and GCC3 domain-containing protein

Our data confirmed the expression of BboGDP1 and -3 proteins on the surface of in vitro-induced sexual stage parasites, while no reaction to *B. bovis* blood stages was detected. In addition, we were able to confirm that antibodies against BboGDP1 and -3 displayed a reactivity against kinetes but not to *B. bovis* blood stages, as reported previously [12]. Collectively, the data demonstrated that one pair of BboGDP, BboGDP1 and -3, was expressed by *B. bovis* tick stages, including sexual stages and kinetes. A previous work in *Plasmodium* demonstrated that sporozoites lacking expression of PCRMP3 and -4 proteins were unable to transmit to the mammalian host [10, 21]. Therefore, knocking out BboGDP genes would be an important step in future studies investigating the function and role of these gene pairs that encode proteins during the parasite life-cycle within the tick vector.

## Conclusions

The data presented herein demonstrated that BboGDP1 and -3 proteins were expressed on the surface of *B. bovis* sexual stages during development and by kinetes. These findings suggest that this pair of BboGDP proteins plays an important role through interactions between parasites and the tick vector. *Babesia bovis* sexual stages and kinetes are free in the lumen tick MG or hemolymph, respectively, and could be targeted by antibodies against these proteins to prevent parasite development within the vector. Therefore, BboGDP1 and -3 are potential candidates for the development of a *B. bovis* transmission blocking vaccines to control the spread of *B. bovis* by tick vectors.

## Data Availability

All data generated or analyzed in this study are included within the article.

## Abbreviations

BboGDP: *Babesia bovis* GCC2/GCC3 domain protein
cDNA: Complementary DNA
6-Cys: 6-Cysteine
GCC: Grip and coiled-coil domain
ISS: Induced sexual stages
MG: Midgut
PBS: Phosphate-buffered saline
PCRMP: *Plasmodium* Cysteine Repeat Modular Protein
PI: Pre-immune
qPCR: Quantitative PCR
XA: Xanthurenic acid
